# Rapid Electrochemical Profiling of Fecal Short-Chain Fatty Acids Using Esterification/Dissociation Fingerprints and Artificial Neural Networks

**DOI:** 10.3390/bios16040223

**Published:** 2026-04-17

**Authors:** Bing-Chen Gu, Guan-Ying Jiang, Ching-Hung Tseng, Yi-Ju Chen, Chun-Ying Wu, Zhi-Xuan Lin, Zhung-Wen Yeh, Chia-Che Wu

**Affiliations:** 1 Semiconductor and Green Technology Doctoral Degree Program, Academy of Circular Economy, National Chung Hsing University, Taichung 40227, Taiwan; d111002602@mail.nchu.edu.tw; 2 Department of Dermatology, Taichung Veterans General Hospital, Taichung 407219, Taiwan; h76183945@gmail.com (G.-Y.J.); yjchenmd@vghtc.gov.tw (Y.-J.C.); 3 Germark Biotechnology Co., Ltd., Taichung 407754, Taiwan; chtzeng@gmail.com; 4 Department of Post-Baccalaureate Medicine, National Chung-Hsing University, Taichung 402202, Taiwan; 5 Institute of Biomedical Informatics, National Yang Ming Chiao Tung University, Taipei 112304, Taiwan; cywu4@nycu.edu.tw; 6 Microbiota Research Center, National Yang Ming Chiao Tung University, Taipei 112304, Taiwan; 7 Health Innovation Center, National Yang Ming Chiao Tung University, Taipei 112304, Taiwan; 8 Ph.D. Program of Interdisciplinary Medicine, National Yang Ming Chiao Tung University, Taipei 112304, Taiwan; 9 Division of Translational Research, Taipei Veterans General Hospital, Taipei 112201, Taiwan; 10 Department of Medical Research, Taichung Veterans General Hospital, Taichung 407219, Taiwan; 11 College of Public Health, China Medical University, Taichung 406040, Taiwan; 12 Department of Mechanical Engineering, National Chung Hsing University, Taichung 40227, Taiwan; sam25236485@gmail.com (Z.-X.L.); rain12369@gmail.com (Z.-W.Y.); 13 i-Center for Advanced Science and Technology, National Chung Hsing University, Taichung 40227, Taiwan

**Keywords:** voltammetric fingerprinting, dual pretreatment (esterification/dissociation), machine-learning-assisted electroanalysis, gut microbiota biomarkers

## Abstract

Short-chain fatty acids (SCFAs) are key biomarkers of gut microbiota activity; however, routine quantification in fecal samples relies largely on chromatography, which is instrument-intensive and throughput-limited chromatography techniques. Herein, we present a rapid machine-learning-assisted electroanalysis platform for SCFAs profiling that integrates a disposable three-electrode planar gold chip with voltammetric fingerprinting and artificial neural network (ANN)-based signal decoupling. To generate orthogonal chemical information and improve the discrimination of structurally similar species, a dual pretreatment strategy combining acid-catalyzed esterification and alkaline dissociation was employed prior to electrochemical analyses. Differential pulse voltammetry (DPV) and cyclic voltammetry (CV) were employed to acquire high-dimensional fingerprints, from which current-, potential-, and area-based descriptors were extracted using a cross-information feature strategy. A hierarchical modeling framework improved total SCFAs prediction by incorporating ANN-predicted propionate and butyrate concentrations as auxiliary inputs. While linear calibration was achievable in standard mixtures, direct linear models performed poorly in real fecal matrices due to strong sample-dependent matrix interference. In contrast, the ANN captured nonlinear relationships among multifeature inputs and suppressed matrix effects. Validation against gas chromatography–mass spectrometry in an independent fecal test cohort (*n* = 30) demonstrated excellent agreement and low prediction errors, with mean absolute error/root mean square error values of 0.063/0.072 mM (propionic acid), 0.029/0.034 mM (butyric acid), and 0.135/0.202 mM (total SCFAs). The DPV/CV acquisition requires only minutes per sample, whereas pretreatment takes 1~3 h depending on the target route but can be performed in parallel for batch processing; thus, overall throughput is determined mainly by batch pretreatment rather than per-sample instrument time. This electrochemical–ANN workflow provides a portable, high-throughput alternative to chromatography for fecal SCFAs profiling in clinical screening and microbiome research.

## 1. Introduction

Short-chain fatty acids (SCFAs) are saturated fatty acids containing fewer than six carbon atoms. In the mammalian gut, these acids are mainly produced by anaerobic intestinal microbiota via the fermentation of dietary fiber and resistant starch, with acetate, propionate, and butyrate as the predominant species. The total SCFAs concentration in the intestinal lumen typically ranges from 20 to 140 mM [1], with these three metabolites accounting for ~90–95% of the total SCFAs pool [2]. The molar ratio of acetate:propionate:butyrate in the colon is commonly reported as ~60:25:15 [3]. SCFAs are critical mediators of host homeostasis and have been linked to the development, prevention, and management of various diseases, including inflammatory bowel disease (IBD), irritable bowel syndrome (IBS), metabolic disorders, and several types of cancers [4,5]. In addition, endogenous SCFA profiles provide valuable insights into diet–microbiota–host metabolic interactions [6]. Among these metabolites, acetate contributes to systemic energy balance and immune modulation [7,8], propionate participates in lipid metabolism and may reduce cardiovascular risk [9,10], and butyrate supports intestinal barrier integrity, suppresses inflammation, and prevents colon carcinogenesis [11]. From a clinical perspective, fecal SCFAs are best interpreted not as a direct proxy for “probiotic counts” but as a functional metabolic readout of microbial fermentation activity and intestinal homeostasis, which can support screening and longitudinal monitoring (e.g., dietary interventions, microbiome-modulating treatments, or disease follow-up) rather than serving as a single definitive diagnostic marker.

At present, SCFAs are predominantly quantified using chromatography-based methods such as gas chromatography (GC) or liquid chromatography (LC) coupled with flame ionization detection (FID) or mass spectrometry (MS) [12,13]. Although these methods are robust, they rely on centralized instrumentation and specialized operators. Moreover, chromatographic separation inherently limits throughput, requiring minutes to tens of minutes per sample for analysis [14,15]. Therefore, rapid and portable assays that require simplified sample preparation and maintain high quantitative accuracy must be developed. In particular, electrochemical sensing platforms that can achieve SCFAs analysis performance comparable to chromatography-based assays remain scarce in practical clinical applications [16].

Electrochemical sensing offers distinct advantages for point-of-care and in-field analysis, including short assay time, portability, and low cost. However, electrochemical signals are often strongly affected by matrix effects in complex biological samples. Coexisting electroactive compounds and background constituents in such samples can obscure target responses and reduce quantitative accuracy [17]. In addition, the direct electro-oxidation of SCFAs typically occurs at relatively high potentials; consequently, many studies employ nanomaterials or other surface-modification strategies to facilitate detection. Although such modifications can enhance sensitivity, they may also introduce coating non-uniformity, stability concerns, and reduced electrode-to-electrode reproducibility, which can compromise reliability in disposable or large-scale screening applications. Accordingly, rather than relying solely on catalytic electrode modification, we emphasize generating differentiable electrochemical fingerprints through targeted solution-phase chemistry and, subsequently, decoupling matrix interference using multifeature learning. For SCFAs detection, studies have demonstrated electrochemical feasibility under controlled conditions, e.g., using poly(3,4-ethylenedioxythiophene) (PEDOT)-modified electrodes [18] and employing linear sweep voltammetry (LSV) to monitor propionate and butyrate used in microbial electrolysis systems [19]. However, these investigations were conducted using standard solutions or simplified models rather than authentic fecal matrices. Impedance-based detection in gut-mimicking systems has indicated that the presence of abundant coexisting analytes in fecal matter can considerably reduce signal-to-noise ratio and hinder reliable quantification in real specimens [20].

To overcome matrix complexity, artificial intelligence (AI) can be integrated with electrochemical fingerprints for decoupling overlapping signatures and suppress interference. Machine learning (ML) techniques can extract subtle concentration-dependent patterns from high-dimensional electrochemical data, thereby improving quantification accuracy in challenging backgrounds. Recent studies have demonstrated the potential of electrochemistry–AI integration for multianalyte decoupling and interference mitigation [21,22]. For instance, ML-assisted quantification has been employed for quantifying food analytes such as caffeine and chlorogenic acid [23] and for biosensing under strong interferent backgrounds such as dopamine detection in the presence of epinephrine [24]. Artificial neural networks (ANNs) can enhance electrochemical biomarker analysis and support the development of cost-effective diagnostic systems [25].

**Figure 1:**
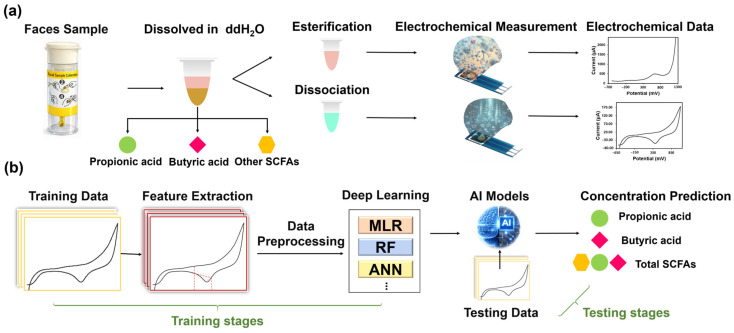
Schematic workflow for quantifying propionate, butyrate, and total SCFAs in fecal samples using dual-route pretreatment, voltammetric fingerprinting, and machine-learning-based signal decoupling. (**a**) Training stage: fecal samples are split into two pretreatment routes (esterification and alkaline dissociation) to generate complementary DPV/CV fingerprints on disposable planar Au chips; fingerprints are converted into engineered descriptors (currents, potentials, and baseline-corrected integrated areas) for model training (MLR/RF/ANN). Dashed red lines indicate the feature windows/points used for extraction. (**b**) Testing stage: the trained model is applied to new fecal samples to decouple matrix-distorted fingerprints and output SCFA concentrations. Arrows denote workflow direction and route branching. Symbol legend: green circle = propionic, pink diamond = butyric, yellow hexagon = acetic; the combined set (green + pink + yellow) represents total SCFAs.

In this study, a dual-signal electrochemical–ANN strategy is proposed for quantifying propionate, butyrate, and total SCFAs in human fecal samples using a disposable three-electrode planar gold chip. As shown in Figure 1, fecal samples are subjected to targeted pretreatment routes that exploit esterification and alkaline dissociation to generate differentiable electrochemical responses. Subsequently, cyclic voltammetry (CV) and differential pulse voltammetry (DPV) are performed to obtain complementary electrochemical fingerprints. Characteristic features extracted from these signals are used to train ML models, such as random forest (RF), multiple linear regression (MLR), and ANNs. The ANN is employed to capture nonlinear relationships and suppress interference resulting from the complex fecal matrix. This strategy is vital for distinguishing propionate and butyrate, which differ by only one methylene group (–CH_2_–) and, therefore, exhibit highly similar chemical behaviors. Finally, the ANN is benchmarked against alternative regression architectures to demonstrate its improved accuracy for SCFAs quantification in real fecal matrices. This AI-enabled electrochemical platform offers a rapid and practical pathway toward SCFAs profiling for clinical gut-health monitoring. During the testing stage, the trained model is applied to new fecal samples to decouple overlapping signatures, particularly for propionate and butyrate, and to output calibrated concentrations of individual acids and total SCFAs. The integration dual-signal acquisition based on esterification and dissociation with AI-driven feature learning (Figure 1) enables rapid SCFAs profiling under strong fecal-matrix interference; this approach also improves reliability relative to conventional linear regression approaches. Notably, the electrochemical readout requires only minutes per sample for DPV/CV acquisition and pretreatment can be performed in batches, enabling higher analytical throughput compared with chromatography-based workflows.

## 2. Materials and Methods

### 2.1. Chemicals and Reagents

All chemicals were of analytical grade and used as received in the experiments. Acetic acid, propionic acid, butyric acid, and potassium hydroxide (KOH) were purchased from Sigma-Aldrich, St. Louis, MO, USA. Phosphate-buffered saline (PBS) and sulfuric acid were obtained from Echo Chemical (Miaoli County, Taiwan). Absolute ethanol (puriss. p.a.) was purchased from Honeywell (Charlotte, NC, USA), and sodium carbonate was obtained from Thermo Fisher Scientific (Waltham, MA, USA). All aqueous solutions were prepared using double-deionized water (ddH_2_O). Unless otherwise specified, experiments were performed at 25 °C ± 2 °C.

### 2.2. Instrumentation and Electrodes

Electrochemical measurements were performed using a portable VBS-100 workstation (VidaBio Technology, Taichung, Taiwan). Unmodified gold SPEs were selected to maximize manufacturability and electrode-to-electrode reproducibility for disposable assays; analytical selectivity was enhanced primarily through dual-route pretreatment and ANN-based signal decoupling rather than catalytic surface modification. Gold-based G3 SPEs (VidaBio Technology, Taichung, Taiwan) comprised a gold working electrode, carbon counter electrode, and on-chip Ag/AgCl reference electrode. The exposed Au working-electrode geometry is defined by a screen-printed insulating layer, yielding a fixed circular area across chips (diameter: 5.5 mm; geometric area: 23.75 mm^2^); therefore, current normalization by electrode area was not required in this study. All measurements used the same SPE model/specification. Prior to use, the gold working electrode was cleaned and activated via UV–ozone treatment λ = 254 nm for 60 s to remove surface organic contaminants. The reference quantification of fecal SCFAs was performed via GC–MS (Shimadzu, Kyoto, Japan) at the Health Technology Center, Chung Shan Medical University (Taichung, Taiwan).

### 2.3. Ethical Approval and Participant Enrollment

The study protocol was approved by the Institutional Review Board of Taichung Veterans General Hospital, Taichung, Taiwan (IRB No. SE23531C). Written informed consent was obtained from all participants prior to enrollment. Participants were recruited from the Department of Dermatology at Taichung Veterans General Hospital between December 2023 and October 2025. Eligible volunteers were 18–65 years of age and were excluded if they were pregnant, planning pregnancy, breastfeeding, or had known pre-existing gastrointestinal diseases.

### 2.4. Fecal Specimen Collection and Preprocessing

Fresh fecal samples were collected and transported to the laboratory in containers with four pre-frozen ice packs. Samples were received within 24 h under refrigerated conditions (4 °C) without preservatives. Upon arrival, each specimen was divided into three aliquots (7.5 g each) and stored at −80 °C. All preprocessing was completed within three days. Frozen aliquots were thawed at room temperature for 30 min, diluted five times with ddH_2_O, and vortex-mixed for 30 s to generate fecal slurries. The slurries were then centrifuged at 3000 rpm at 4 °C for 10 min, and supernatants from the three aliquots were pooled and stored at −80 °C for electrochemical analysis and GC–MS measurements. To account for strong matrix effects during model development, two preparation streams were used, as follows: (i) a centrifuged stream (supernatant) to improve signal consistency and (ii) a noncentrifuged stream, in which slurries were vortexed for 10 s and analyzed directly to capture background and noise signatures for ANN training.

### 2.5. Reaction Mechanisms and Procedures

Two complementary chemical pathways were employed to differentiate structurally similar SCFAs: esterification and alkaline dissociation. Acid-catalyzed esterification (Equation (1)) converts carboxylic acids to their corresponding ethyl esters, whereas alkaline dissociation pathway (Equation (2)) generates carboxylate ions under basic conditions:

(1) $$R-COOH+R^{\prime }-OH\overset{H^{+}}{\to }R-COOR^{\prime }+H_{2}O\;(acid‐catalyzed)$$

(2) $$R-COOH\to R-COO^{-}+H^{+}$$

**Figure 2:**
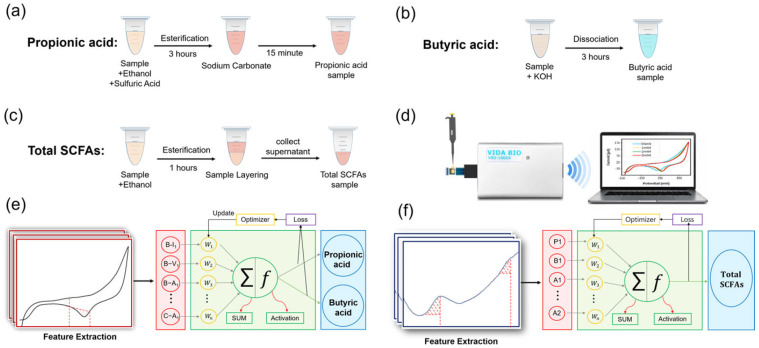
Experimental setups and modeling workflow for electrochemical quantification of SCFAs. (**a**–**c**) Pretreatment and measurement configurations for propionic acid, butyric acid, and total SCFAs, respectively; (**d**) overall sensing procedure using a portable electrochemical analyzer to record voltammetric fingerprints, followed by feature extraction and data preprocessing; (**e**) ANN model for the simultaneous prediction of propionic and butyric acids.; (**f**) ANN model for the prediction of total SCFAs. Dashed red lines indicate the feature windows/points used for extraction.

Propionic acid (esterification route; Figure 2a): 1.0 mL of sample was mixed with 1.0 mL of ethanol and 30 μL of sulfuric acid (98%) and reacted for 3 h. The reaction mixture was neutralized with 2 M $Na_{2}CO_{3}$ for 15 min at room temperature.

Butyric acid (dissociation route; Figure 2b): 1.0 mL of sample was mixed with 1.0 mL of 0.6 mM KOH and reacted for 3 h at room temperature.

Total SCFAs (alternative esterification route; Figure 2c): 1.2 mL of sample was mixed with anhydrous ethanol at a 2:1 (*v*/*v*; sample: ethanol) ratio and reacted for 1 h at room temperature. Then, DPV and CV were performed within 3 min for each sample.

Pretreatment is the time-dominant step (1–3 h depending on route); however, these reactions are compatible with parallel batch processing, allowing multiple samples to be prepared simultaneously rather than sequentially.

### 2.6. Electrochemical Protocols and Feature Extraction

As summarized in Figure 2d, pretreated samples were analyzed to obtain voltammetric fingerprints, followed by feature extraction and preprocessing for model training. For each measurement, 200 μL of prepared sample was deposited onto the SPE. All voltammetric measurements were performed on single-use disposable Au SPEs; each chip was used for only the first DPV or CV scan and then discarded, using only the first DPV/CV scan per chip, and that each sample was measured in triplicate using fresh electrodes. All subsequent measurements were carried out with a fresh electrode to minimize drift associated with repeated cycling, Au surface-history effects, and matrix fouling. During method optimization, both CV and DPV were screened for each pretreatment route to examine the overall electrochemical behavior and to identify feature windows that were robust and concentration-dependent; for routine analysis and ANN consistency, the final protocol records one voltammogram per pretreatment route (DPV for esterification routes and CV for the dissociation route) to minimize acquisition time.

1. Propionic acid (esterification route): DPV was recorded from −600 to 2000 mV (PH 150 mV; PW 2 ms; SH 5 mV). After esterification-based pretreatment, DPV provided clearer concentration-dependent fingerprints with reduced capacitive background compared with CV, enabling reliable extraction of current/area descriptors from predefined potential windows.
2. Butyric acid (alkaline dissociation route): CV was recorded from −600 to 1100 mV at 200 mV s^−1^. Under dissociation pretreatment, the informative response manifested as a broader, repeatable CV fingerprint in the anodic region; the descriptor at 175 mV showed stable monotonic behavior, whereas DPV exhibited less distinct concentration-dependent variation under the same conditions.
3. Total SCFAs (esterification route): DPV was recorded from −600 to 1200 mV (PH 100 mV; PW 50 ms). Similar to the propionate route, DPV offered improved feature visibility and repeatability relative to CV after esterification pretreatment.

Electrochemical descriptors used for modeling included peak/characteristic currents (I), characteristic potentials (V), and baseline-corrected integrated areas (A). Currents and potentials are reported in μA and mV, respectively, and baseline-corrected area features are reported in μA·mV. To enhance selectivity in complex matrices, a cross-information strategy was employed: the propionic and butyric acid models used a 10-feature input vector (six propionate-related and four butyrate-related descriptors), whereas the total SCFAs model used a 7-feature input vector (five DPV descriptors plus auxiliary inputs from the propionate/butyrate models). DPV/CV acquisition requires only minutes per sample (typically ≤3 min total to record the DPV/CV fingerprints for the three targets), whereas overall turnaround time is determined mainly by the pretreatment steps (Section 2.5), which can be performed in parallel for batch processing. For a batch of N samples, the workflow (please see Section S1) can be approximated as T_EC_ ≈ 3.25 h + (3 min × N) under the current protocol. Accordingly, the calibration models are intended for interpolation within the validated working range; specimens outside this range can be brought into range by appropriate dilution, and extension of the calibration range (e.g., adding higher-concentration standards) will be pursued in future work as needed. Potential offsets due to uncompensated resistance were estimated to be minor at the μA current level (iR ≈ I × Rs); an EIS-based chip quality-control dataset is provided in Figure S1 to support the order-of-magnitude assessment, and ANN features were extracted from baseline-corrected windows designed to be robust to small potential shifts.

### 2.7. ANN Modeling and Signal Decoupling

ANN models (Figure 2e,f) were implemented in TensorFlow to decouple overlapping SCFAs signatures and suppress matrix interference. A multilayer perceptron (MLP) architecture was employed, which comprised an input layer, hidden layers, and an output layer for concentration prediction [26]. The rectified linear unit (ReLU) activation function was applied to introduce nonlinearity [27,28,29]. To improve numerical stability and avoid scale imbalance among descriptors, feature-wise min–max normalization was applied using training-set parameters only. Details of leakage-free normalization, hyperparameter screening, and cross-validation-based architecture selection are provided in Supplementary Methods S2 and Tables S1 and S2.

For propionic acid (DPV), the following six features were extracted from centrifuged and noncentrifuged samples: currents at −30 mV (B_I1, B_I2; centrifuged), potentials at 1000 μA (B_V1, B_V2; noncentrifuged), and integrated areas from −230 to −30 mV (B_A1, B_A2; noncentrifuged). For butyric acid (CV), four features were extracted: oxidation and reduction peak currents (CI_1, CI_2), potential at 10 μA (C_V1), and integrated area from 500 to 110 mV (C_A1). These parameters were combined into 10-feature vectors (cross-information) for individual prediction of propionic and butyric acids.

**Figure 3:**
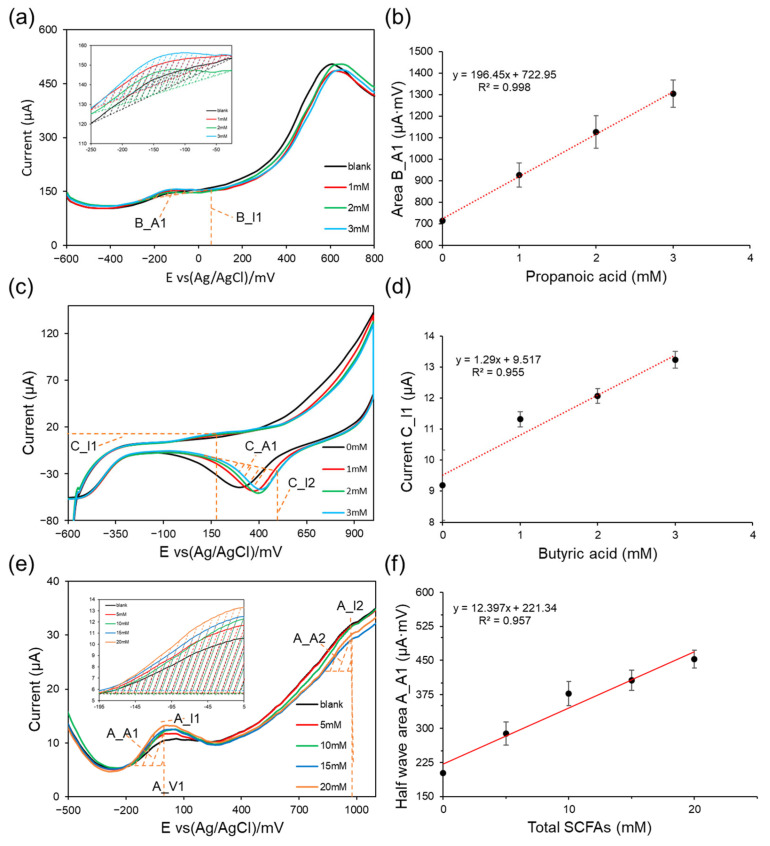
Voltammetric fingerprints and feature-based calibration of SCFAs in standard mixtures under fixed-background compositions: (**a**) DPV responses for propionic acid after esterification-based pretreatment, recorded while holding acetate and butyrate constant and varying propionic acid concentration; (**b**) Propionic acid calibration derived from the integrated DPV area (B_A1) within the −230 to −30 mV window (714.68 ± 2.11, 926.66 ± 56.33, 1126.66 ± 75.25, 1303.02 ± 63.56 μA·mV for 0, 1, 2 and 3 mM, respectively, n = 3); (**c**) CV responses for butyric acid after alkaline dissociation pretreatment, recorded while holding acetate and propionate constant and varying butyric acid concentration; (**d**) Butyric acid calibration derived from the CV current (C_I1) measured at 175 mV (9.19 ± 1.13, 11.32 ± 0.25, 12.07 ± 0.24, 13.24 ± 0.27 μA for 0, 1, 2 and 3 mM, respectively, n = 3); (**e**) DPV responses for total SCFAs after esterification-based pretreatment under fixed propionate and butyrate concentrations while varying acetate to modulate total SCFAs; (**f**) Total SCFAs calibration derived from the integrated DPV feature (A_A1) within the designated potential window (201.35 ± 0.23, 289.17 ± 25.69, 376.94 ± 37.87, 406.48 ± 22.04, 452.62 ± 19.86 μA·mV for 0, 5, 10, 15 and 20 mM, respectively, n = 3). Insets highlight the potential regions used for feature extraction. Integrated areas were obtained by baseline-corrected integration and are reported in μA·mV.

For total SCFAs (DPV; Figure 3e), five DPV-derived features were defined from two characteristic regions. In the first region (−200 to 100 mV), A_I1 was defined as the characteristic peak current (maximum current) within this window, and A_V1 was defined as the corresponding peak potential at which A_I1 occurs. The associated area descriptor A_A1 was calculated as the left half-wave area of the waveform, obtained by integrating the DPV signal from −200 mV to A_V1 (i.e., up to the peak potential). In the high-potential region, A_I2 was defined as the characteristic current at 985 mV, and A_A2 was defined as the left half-wave area obtained by integrating the waveform from 800 to 985 mV. Currents are reported in μA, potentials in mV. Integrated-area features (e.g., B_A1, A_A1, and A_A2) were calculated by baseline-corrected numerical integration of the DPV trace over the specified potential window and are reported in μA·mV.

Based on cross-validated hyperparameter screening (Table S1) and train–validation/test consistency (Table S2), the final propionate/butyrate ANN used one hidden layer with 4000 neurons, ReLU activation, dropout = 0, Adam optimizer (learning rate 1 × 10^−4^), Huber loss, batch size 50, and 5000 epochs. The total SCFAs ANN used one hidden layer with 1000 neurons, ReLU activation, dropout = 0.1, Nadam optimizer (learning rate 1 × 10^−4^), Huber loss, batch size 30, and 5000 epochs.

The propionate/butyrate model was developed using 102 cases (72 training cases, including 18 authentic fecal samples; and an independent test set of 30 authentic fecal samples). The total SCFAs model was developed using 186 cases (156 training cases, including 30 authentic fecal samples, and the same 30 authentic fecal samples used as an independent, sample-matched test set across analytes). The dataset composition is summarized in Table 1. Model performance was evaluated against GC–MS reference measurements using the coefficient of determination (R^2^), mean absolute error (MAE), and root mean square error (RMSE) [30,31]. Predictions were additionally summarized by agreement rates within preset deviation thresholds (e.g., ±5% and ±10%), as reported in Section 3.3. In addition, model stability and overfitting risk were assessed using five-fold cross-validation as described in Section 2.8.

Because acetate/propionate/butyrate dominate the fecal SCFAs pool, acetate was reported as a derived mass-balance estimate:

[Acetate]_est_= [Total SCFAs] − [Propionate] − [Butyrate] (3)

This value should be interpreted as an acetate-dominant residual that may also reflect minor SCFAs included in “total SCFAs”, and it propagates the prediction uncertainties of the three contributing terms; direct acetate validation will be addressed in future work.

### 2.8. Statistical Analysis and Model Validation

Pearson correlation analysis was performed to quantify linear associations between individual electrochemical features and SCFAs concentrations, supporting feature screening and interpretation [32,33]. MLR was used as a baseline model to evaluate linear explainability of SCFAs variance and to benchmark nonlinear models [34].

Model robustness and potential overfitting was evaluated via five-fold cross-validation (k = 5) [35]. The available dataset was partitioned into five subsets, and iterative training–validation cycles were performed such that each subset served once as the validation set. Model stability was assessed by comparing fold-to-fold loss profiles and performance consistency. When substantial fold-to-fold variance suggested possible overfitting or strong outlier influence, additional sensitivity analyses were performed by repartitioning the training data using smaller holdout fractions (5–10%) to identify instability sources and refine model settings.

To strengthen agreement assessment against the reference method, method-comparison statistics were performed on the independent fecal test set benchmarked against GC–MS. In addition to R^2^/MAE/RMSE, agreement was evaluated using Bland–Altman analysis (mean bias and limits of agreement) and uncertainty estimation via 95% confidence intervals (e.g., bootstrap confidence intervals for RMSE and mean bias). Paired hypothesis testing (paired *t*-test and/or Wilcoxon signed-rank test, two-sided α = 0.05) was used to assess whether systematic bias differed from zero. The final performance reported in Table 2 was obtained from the independent fecal test set benchmarked against GC–MS. Bland–Altman plots are provided in Figures S4–S6, and the corresponding 95% confidence intervals for RMSE and mean bias are summarized in Table S3.

## 3. Results and Discussion

The proposed dual-route electrochemical–ANN strategy for SCFAs quantification was evaluated. The analysis progressed from feature identification and calibration in standard solutions to model training and validation in complex fecal matrices. First, concentration-dependent voltammetric descriptors were established under controlled backgrounds. Then, how multivariate learning suppressed matrix interference to enable accurate prediction in real specimens was demonstrated. In this analysis, extensive chromatographic separation was replaced with targeted chemical pretreatment to generate characteristic electrochemical fingerprints. Then, the ANN was employed to computationally suppress matrix interference and decouple overlapping signals.

### 3.1. Analytical Performance in Standard Solutions

First, the electrochemical fingerprints of propionic acid, butyric acid, and total SCFAs in standard mixtures were characterized to identify robust, concentration-dependent features and establish baseline calibration relationships under controlled background conditions. For clarity and reproducibility, the extracted feature windows are highlighted by insets in Figure 3, and all calibration points are reported as mean ± SD from three independent measurements (n = 3). Figure 3 summarizes the concentration-dependent voltammetric responses and corresponding feature-based calibrations. For each series, a 0 mM target (blank) was processed under identical background composition and pretreatment, and area features were calculated by baseline-corrected integration (μA·mV). Although these descriptors exhibit good linearity in standards, fecal matrices introduce sample-dependent (often nonlinear) distortions that cannot be corrected by single-feature calibration; therefore, richer DPV/CV fingerprint information is leveraged for ANN-based signal decoupling in the next section.

**Figure 4:**
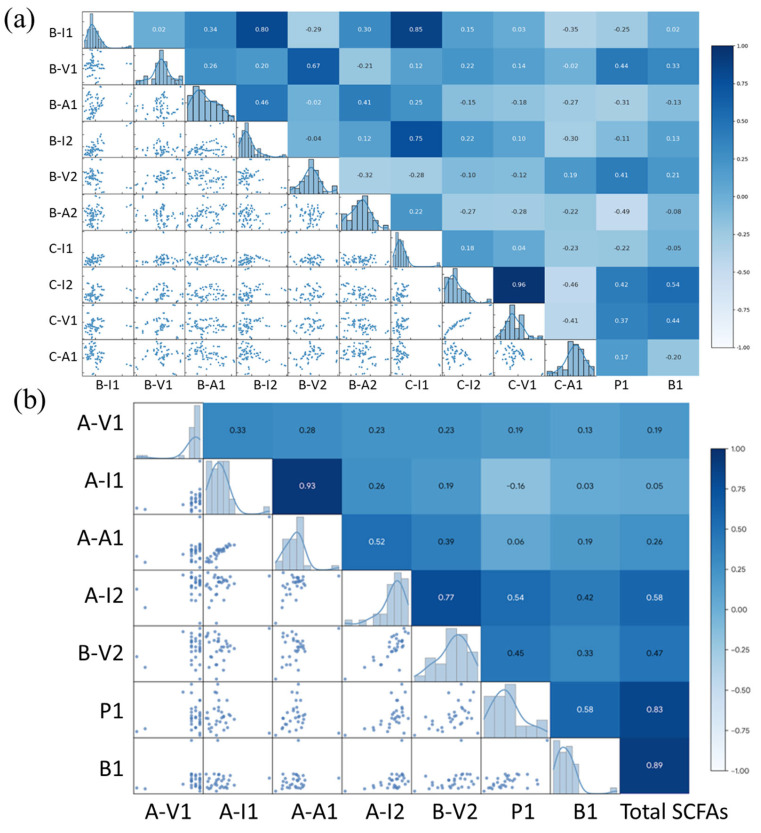
Pearson correlation analysis of electrochemical features used for SCFAs quantification models: (**a**) Correlation matrix (Pearson’s r) for the feature set used in propionic/butyric acid models; (**b**) Correlation matrix (Pearson’s r) for the feature set used in the total SCFAs model. Upper triangles report correlation coefficients, diagonal panels show feature distributions, and lower triangles show pairwise scatter plots; color intensity reflects the strength and direction of correlation.

For consistency with the feature notation used in Section 2.7 and Figure 4, the extracted descriptors are labeled using a prefix letter to indicate the target model and a subscript to indicate the feature type. Specifically, B_* denotes features used for propionic acid prediction, C_* denotes features used for butyric acid prediction, and A_* denotes features used for total SCFAs prediction. The subscripts I, V, and A represent current-based, potential-based, and area-based descriptors, respectively, while the trailing index (e.g., 1 and 2) distinguishes multiple features of the same type. Thus, I-features correspond to characteristic currents measured at selected potentials (or peak currents), V-features correspond to characteristic potentials (e.g., peak potential or the potential at a specified current threshold), and A-features correspond to integrated areas within predefined potential windows capturing the fingerprint region of interest (e.g., B_A1 for the propionate DPV area from −230 to −30 mV; C_I1 for the butyrate CV current feature at 175 mV; and A_I1/A_V1/A_A1/A_I2/A_A2 for total SCFAs DPV descriptors as defined in Figure 3e).

To obtain propionic-acid fingerprints, the concentrations of acetic acid and butyric acid were fixed at 6 and 2 mM, respectively, while propionic acid was varied from 0 to 3 mM (0, 1, 2, and 3 mM). After esterification-based pretreatment, DPV measurements revealed a distinct fingerprint feature within approximately −230 to −30 mV (Figure 3a). The integrated area over this window (B_A1) increased monotonically with propionic acid concentration and showed a linear relationship, enabling construction of a calibration curve (Figure 3b). As shown in Figure 3b, the baseline-corrected integrated area feature (B_A1; μA·mV) is reported as 714.68 ± 2.11, 926.66 ± 56.33, 1126.66 ± 75.25, 1303.02 ± 63.56 μA·mV for 0, 1, 2, and 3 mM (n = 3) and exhibits clear separation between concentration levels, indicating that the concentration-dependent change is reproducible and exceeds replicate variability under fixed-background conditions. In addition to B_A1, complementary B_I- and B_V-type descriptors (currents and potentials derived from the same DPV trace, as defined in Section 2.7) can be extracted for subsequent multifeature modeling. Under these controlled conditions, the selected DPV feature was predominantly governed by propionic acid concentration, indicating sensitivity and selectivity when other SCFAs were held constant.

To obtain butyric-acid fingerprints, the concentrations of acetic acid and propionic acid were fixed at 6 and 2 mM, respectively, while butyric acid was varied from 0 to 3 mM. After alkaline dissociation pretreatment, CV profiles exhibited a concentration-dependent change in the anodic-region response, with a representative variation observed around 172–175 mV (Figure 3c). The extracted descriptor—CV current at 175 mV (C_I1)—increased linearly with concentration, enabling construction of a calibration equation (Figure 3d). Because Au electrodes can show surface/oxide-related contributions and scan-history effects—particularly in the broad cathodic region near 350–400 mV—we treat the CV trace as a fingerprint-based interfacial response rather than assigning that region to a specific faradaic butyrate redox peak. Here, C_I, C_V, and C_A denote current-, potential-, and area-based descriptors derived from the CV fingerprint (Section 2.7). These results indicate that C_I1 provides a reproducible concentration-dependent fingerprint for butyrate quantification under fixed-background conditions.

To characterize total SCFA fingerprints, the concentrations of propionic acid and butyric acid were fixed at 2 mM each, while acetate was varied (0, 5, 10, 15, and 20 mM) to modulate total SCFA levels. After esterification-based pretreatment, DPV measurements showed that the response near the main feature region increased with increasing acetate (Figure 3e). As illustrated in Figure 3e, two DPV regions were quantified for total SCFAs. In the low-potential window (−200 to 100 mV), A_V1 denotes the characteristic peak potential and A_I1 denotes the corresponding peak current; A_A1 is the baseline-corrected left half-wave area integrated from −200 mV to A_V1 (units: μA·mV). In the high-potential region, A_I2 is defined as the characteristic current at 985 mV, and A_A2 is the baseline-corrected left half-wave area integrated from 800 to 985 mV (μA·mV). The calibration in Figure 3f was constructed using the integrated DPV feature A_A1, which exhibited a linear dependence on concentration under the controlled standard-mixture condition.

Figure 3 demonstrates that each pretreatment–voltammetry combination yields a reproducible, concentration-dependent electrochemical descriptor in standard solutions. Together, these standard-mixture results confirm statistically repeatable feature extraction (mean ± SD, n = 3), but the resulting single-feature calibrations are not directly transferable to fecal specimens because matrix-dependent baseline shifts, waveform deformation, and nonlinear interference require multifeature ANN-based decoupling (Section 3.2).

### 3.2. ANN Model Performance and Matrix Interference Suppression

To address the reduced selectivity and increased variability observed in fecal samples, ANN models were developed to integrate multiple electrochemical descriptors and suppress matrix-induced distortions. Rather than relying on a single calibration feature, the propionic-acid and butyric-acid models employed an expanded 10-feature input vector (including cross-information descriptors) to capture nonlinear relationships among voltammetric features and background interference. For total SCFAs, prediction performance was further improved using a hierarchical input design, in which the ANN-predicted propionic acid (P1) and butyric acid (B1) concentrations were incorporated as auxiliary variables. This increased the total SCFAs input dimensionality from 5 to 7 features and improved predictive performance (R^2^ = 0.99929). Collectively, this structure helps account for sample-to-sample variability and the variable contributions of individual acids (and minor species) that cannot be reliably separated using any single electrochemical descriptor in complex fecal backgrounds.

Pearson correlation analysis (Figure 4) was performed to examine redundancy and complementarity among the extracted features prior to constructing the model. For propionic and butyric acids (Figure 4a), several feature pairs showed strong positive or negative correlations, indicating partial redundancy. In contrast, other pairs exhibited weak correlations and thus provided complementary information beneficial for multivariate learning. For total SCFAs (Figure 4b), the correlation results differed and was consistent with distinct pretreatment routes and signal contributions. In addition, clusters of strongly correlated features suggested redundancy, whereas weakly correlated features supported the use of a multifeature ANN model to enhance robustness.

Figure S2 shows parity plots for both training and test sets to demonstrate model fitting and generalization. The ANN achieved strong agreement with reference values for propionic acid (training: R^2^ = 0.994, RMSE = 0.087 mM, and MAE = 0.064 mM; test: R^2^ = 0.998, RMSE = 0.057 mM, and MAE = 0.050 mM) and butyric acid (training: R^2^ = 0.998, RMSE = 0.035 mM, and MAE = 0.028 mM; test: R^2^ = 0.999, RMSE = 0.029 mM, and MAE = 0.025 mM), indicating good generalization with limited overfitting. For total SCFAs, similarly strong agreement was observed (training: R^2^ = 0.999, RMSE = 0.116 mM, and MAE = 0.282 mM; test: R^2^ = 0.999, RMSE = 0.099 mM, and MAE = 0.269 mM). The larger absolute errors observed for total SCFAs are expected because total SCFAs concentrations spanned a wider range and represented an integrated signal contributions from various acids. Subsequent to establishing the feature design and confirming stable training–test behavior, the ANN predictions were validated against GC–MS reference measurements using an independent set of human fecal samples to assess the real-world analytical performance of the model.

### 3.3. Validation with Human Fecal Samples

The practical utility of the electrochemical–ANN workflow was evaluated using independent test cohort of 30 human fecal samples. To illustrate the real-matrix signal characteristics, representative raw DPV/CV fingerprints from three clinical fecal samples are provided in the Supplementary (Figure S3), together with the corresponding feature windows used for ANN input. The predicted concentrations of propionic acid, butyric acid, and total SCFAs were benchmarked against GC–MS reference measurements (Table 2). Across a broad concentration range (total SCFAs: 1.32–21.32 mM), ANN predictions closely matched the GC–MS values and most samples exhibited only minor relative deviations. Specifically, agreement within ±5% was achieved for 27 among 30 samples for total SCFAs (90%), 26 among 30 samples for propionic acid (86.7%), and 24 among 30 for butyric acid (80.0%). The largest percentage deviations were primarily observed at low concentrations, where small absolute differences corresponded to larger relative errors (e.g., propionic acid in sample 15 and butyric acid in sample 14). With n = 30 independent fecal samples, the mean prediction errors are estimated with bounded uncertainty (95% bootstrap CI for RMSE: 0.037–0.071 mM for propionic acid, 0.020–0.038 mM for butyric acid, and 0.122–0.269 mM for total SCFAs), supporting that the observed agreement with GC–MS reflects generalizable performance rather than sampling noise. Overall, these results demonstrated the feasibility of ANN-assisted electrochemical sensing as a feasible and practical approach for estimating SCFAs concentrations in real fecal matrices.

**Figure 5:**
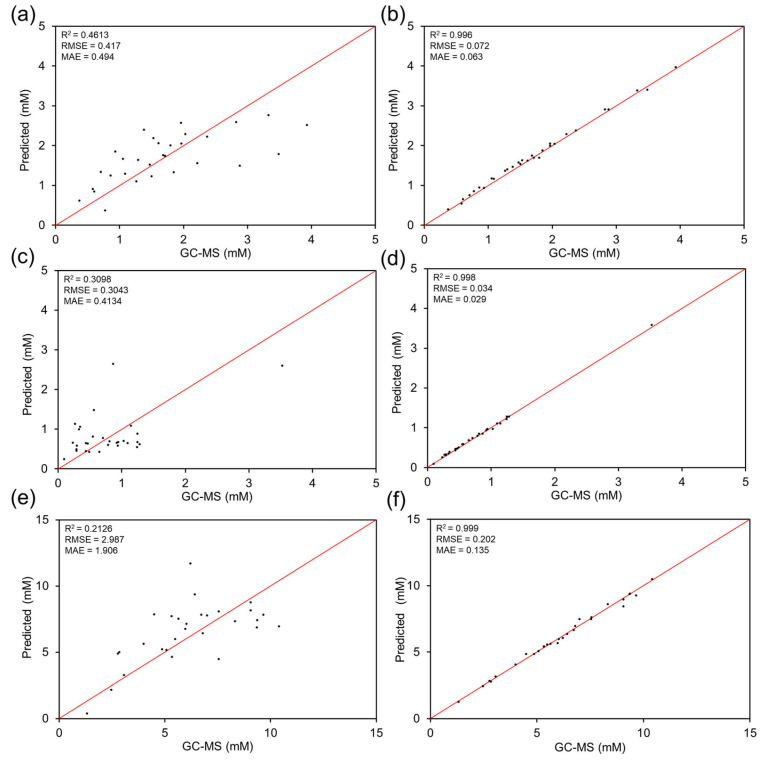
Comparison of MLR and ANN models for SCFAs quantification in human fecal samples using GC–MS as the reference method. Parity plots show predicted concentrations versus GC–MS measurements for (**a**) propionic acid (MLR), (**b**) propionic acid (ANN, test set), (**c**) butyric acid (MLR), (**d**) butyric acid (ANN, test set), (**e**) total SCFAs (MLR), and (**f**) total SCFAs (ANN, test set). The red diagonal line denotes ideal 1:1 agreement; R^2^, RMSE, and MAE are reported in each panel.

As a baseline comparison, MLR and correlation-based analysis were also evaluated to determine whether linear models could explain variability across fecal samples. In real matrices, MLR exhibited poor predictive performance (R^2^ ≈ 0.21–0.46; Figure 5a–e), consistent with nonlinear and sample-dependent interference effects that could not be captured via simple linear mapping. In contrast, ANN predictions maintained near-ideal agreement with GC–MS reference measurements (R^2^ > 0.996 across all targets). Correlation analysis (Figure 4) further revealed that while certain individual features contributed strongly to prediction, a multifeature architecture that integrates complementary descriptors is required to achieve optimal predictive performance. This rationale also supports inclusion of cross-information variables such as the use of B1 as an auxiliary predictor in the total SCFAs model. To contextualize these results and clarify the advantages of nonlinear learning, the predictive performance of the ANN model was compared with those of conventional regression and alternative machine learning architectures using parity plots and aggregate error metrics. Notably, the test cohort was not used for model tuning.

Bland–Altman analysis further supported method agreement (Figures S4–S6), showing small mean biases of −0.015 mM (propionic acid), −0.015 mM (butyric acid), and 0.005 mM (total SCFAs). The 95% limits of agreement were −0.119 to 0.090 mM for propionic acid, −0.065 to 0.035 mM for butyric acid, and −0.396 to 0.406 mM for total SCFAs, suggesting no pronounced systematic offset across the measurement range. Bland–Altman analysis (Figures S4–S6) together with bias testing and RMSE confidence intervals (Table S3) indicated no statistically significant systematic bias for total SCFAs (bias = 0.005 mM; *p* = 0.894/0.577) or propionic acid (bias = −0.015 mM; *p* = 0.144/0.289), whereas butyric acid showed a small but statistically significant negative bias (bias = −0.015 mM; *p* = 0.0027/0.0031), with narrow 95% limits of agreement supporting overall method agreement.

In clinical contexts, fecal SCFAs concentrations are best viewed as a functional metabolic readout of microbial fiber fermentation and intestinal homeostasis rather than a direct surrogate for “probiotic counts”. Practically, the measured values (propionate, butyrate, and total SCFAs, with acetate reported as a derived estimate) can be interpreted as a compact biomarker panel to support screening and longitudinal monitoring, where within-subject trends may be more informative than a single time point. For example, persistently low fecal butyrate or a shift in the balance among acetate/propionate/butyrate has been associated in the literature with reduced barrier-supportive fermentation activity and may be observed during active intestinal inflammation, dietary fiber insufficiency, or microbiome-disrupting exposures. Accordingly, the proposed platform is positioned as a practical, portable tool for large-cohort screening or follow-up measurements, with GC–MS remaining the confirmatory reference when definitive quantification or full speciation is required. Establishing population reference intervals, clinically actionable thresholds, and performance across broader dietary/clinical subgroups will be necessary for future diagnostic deployment and is a key direction of ongoing work.

### 3.4. Comparative Analysis of ML Architectures

Finally, the ANN model was compared with baseline MLR and an RF model to quantify the advantage of nonlinear, multifeature learning for SCFAs prediction in fecal matrices. Here, MLR represents a simple linear baseline, whereas RF serves as a representative nonlinear ensemble model with strong performance in many small-to-medium tabular datasets. This comparison allows us to distinguish whether the improvement arises merely from introducing nonlinearity (RF) or from learning higher-order feature interactions and matrix-dependent distortions more effectively (ANN). Figure 5 shows a direct comparison of MLR and ANN performances using parity plots (predicted vs. GC–MS measurements), where the diagonal line represents ideal 1:1 agreement. For propionic acid, the MLR predictions showed moderate agreement with GC–MS reference measurements (R^2^ = 0.4613, RMSE = 0.417, and MAE = 0.494; Figure 5a), whereas the ANN model considerably reduced data scatter and prediction error (R^2^ = 0.996, RMSE = 0.072, and MAE = 0.063; Figure 5b). For butyric acid, MLR predictions exhibited weak correlation with GC–MS reference measurements (R^2^ = 0.3098, RMSE = 0.3043, and MAE = 0.4134; Figure 5c), whereas the ANN achieved near-ideal agreement (R^2^ = 0.998, RMSE = 0.034, MAE = 0.029; Figure 5d). For total SCFAs, MLR again performed poorly (R^2^ = 0.2126; Figure 5e), whereas the ANN model predictions were highly accurate (R^2^ = 0.999, RMSE = 0.202, MAE = 0.135; Figure 5f).

To further benchmark model selection, the performances of MLR, RF, and ANN were compared using MAE, RMSE, and accuracy (Table 3). For propionic acid, the ANN model achieved the best performance (MAE = 0.063, RMSE = 0.072, and accuracy = 96.66%), outperforming RF (MAE = 0.100, RMSE = 0.142, and accuracy = 90%) and considerably exceeding MLR (accuracy = 20.1%). For butyric acid, the ANN model again achieved the highest accuracy (96.66%) with the lowest errors (MAE = 0.029 and RMSE = 0.034), whereas RF showed intermediate performance and MLR was ineffective (accuracy = 10%). For total SCFAs, the ANN model delivered the best overall performance (MAE = 0.135, RMSE = 0.202, and accuracy = 90.0%), whereas the accuracy of RF decreased (43.3%). Notably, although RF improved markedly over MLR for propionic acid (Table 3), its performance degraded for butyric acid and total SCFAs, indicating limited robustness to sample-dependent matrix shifts and cross-analyte interactions in fecal specimens. In contrast, the ANN consistently achieved the lowest MAE/RMSE and the highest accuracy across all targets, supporting its superior ability to model nonlinear, multifeature relationships under complex fecal-matrix interference. Therefore, the ANN was selected as the final model because it provided the most reliable and target-consistent performance across individual acids and total SCFAs, rather than excelling only for a single analyte. These findings confirmed that modeling high-dimensional, nonlinear electrochemical fingerprints is essential for the reliable quantification of SCFAs in fecal matrices and that the ANN model provided the most robust performance among the evaluated architectures. The analysis of parity plots and model-comparison metrics confirmed that linear approaches are inadequate in real, complex fecal backgrounds, whereas the ANN model consistently achieved the lowest error and highest accuracy. This highlights the importance of employing multivariate nonlinear modeling for practical SCFAs quantification in fecal matrices. Importantly, ANN selection was guided not only by test-set accuracy but also by cross-validation stability and train–validation consistency (Section 2.8), mitigating the risk that improved performance reflects overfitting.

Overall, the results indicate that although SCFAs signals can be quantified using simple linear descriptors in standard solutions, multifeature, nonlinear modeling is required for their reliable quantification in fecal matrices. The proposed ANN model provided the most consistent accuracy and robustness across individual acids and total SCFAs, confirming its suitability for rapid and practical fecal SCFAs profiling. In addition to its high accuracy, this model offers practical advantages in terms of throughput because the electrochemical readout requires only minutes per sample for DPV and CV acquisition. This makes it suitable for high-throughput fecal SCFAs profiling. Although amperometry can offer high sensitivity in relatively simple matrices, we prioritized fingerprint-based DPV/CV because the richer waveform information (e.g., peak position, shape, intensity, and integrated area) is essential for ANN training and improves robustness against fecal-matrix interference.

To contextualize the present workflow relative to published rapid/electrochemical SCFAs methods, Table S4 summarizes representative approaches in terms of analyte scope, matrix, pretreatment, throughput, portability, and validation level. Prior electrochemical studies often demonstrate feasibility in controlled solutions or culture media, whereas comprehensive validation in authentic fecal matrices remains limited. By integrating dual-route pretreatment (esterification/dissociation) with voltammetric fingerprinting and ANN-based signal decoupling, the proposed platform enables multi-analyte profiling with minute-scale electrochemical readout and GC–MS-benchmarked performance in an independent human fecal test cohort (n = 30).

## 4. Conclusions

In this study, a rapid electrochemical strategy was proposed for quantifying SCFAs in human fecal samples by integrating a disposable planar gold three-electrode chip with ANN-based signal decoupling. The proposed workflow combined two complementary pretreatment routes, i.e., esterification and alkaline dissociation, to generate orthogonal voltammetric fingerprints (DPV and CV), thereby strengthening SCFAs-specific features prior to modeling. This dual-route design is crucial for discriminating propionate and butyrate, which differ by only one methylene group and therefore produce highly overlapping electrochemical responses in complex biological matrices.

Using standard solutions, concentration-dependent electrochemical descriptors were established. The results indicated that direct linear calibration was not sufficient for quantifying SCFAs in real fecal samples due to strong matrix effects and sample-to-sample variability. Compared with conventional MLR, the ANN model effectively learned nonlinear relationships among multifeature inputs, suppressed background interference, and provided robust prediction across authentic specimens. When benchmarked against GC–MS reference measurements, the ANN achieved near-ideal agreement (R^2^ > 0.99) for propionate, butyrate, and total SCFAs. In addition, acetate concentrations could be estimated by mass-balance subtraction considering that acetate, propionate, and butyrate dominated the SCFAs pool. Comparative evaluation against alternative machine-learning methods further confirmed that the ANN model delivered the most consistent performance, achieving lower MAE and RMSE values and higher accuracy compared with MLR and RF models.

In practical use, the electrochemical readout requires only minutes per sample for DPV/CV acquisition, while the overall turnaround time is dominated by pretreatment (approximately 1–3 h, depending on the target route). Importantly, these pretreatment steps are compatible with parallel batch processing, so for multi-sample analysis the total time is governed primarily by the batch pretreatment duration plus minute-scale per-sample acquisition, rather than sequential long instrument runs. While GC–MS remains the gold standard for SCFAs quantification due to its high sensitivity and specificity, its high instrument cost, maintenance burden, and operational complexity may limit widespread implementation in large-cohort or decentralized settings. Accordingly, we consider GC–MS as the confirmatory backup method for definitive SCFA quantification when the highest accuracy is required (e.g., borderline cases or out-of-range specimens). By contrast, the proposed platform requires substantially lower instrument investment and maintenance costs than conventional GC–MS systems and is, therefore, well suited for large-scale screening and longitudinal monitoring applications.

While the proposed electrochemical–ANN workflow enables rapid, portable screening, GC–MS remains the confirmatory backup method for definitive SCFA quantification when the highest accuracy is required (e.g., borderline cases or out-of-range specimens). Limitations of the current workflow include the requirement for two pretreatment routes and the dependence of model generalization on training-data representativeness; optional electrode engineering and further assay integration may further enhance robustness without sacrificing reproducibility. Future work will focus on expanding the fecal cohort across broader dietary and clinical variability, improving model transferability across instruments and electrode batches, and streamlining the assay toward a more integrated single-chip implementation, as well as extending the framework to additional fecal metabolites of clinical relevance. In addition, future work will shorten and/or automate the pretreatment steps through kinetic optimization (e.g., reagent ratio, temperature, and mixing control) and batch-compatible or cartridge-based workflows, thereby reducing end-to-end turnaround time while preserving the minute-scale DPV/CV acquisition. Finally, although robustness is supported here by GC–MS-benchmarked validation on authentic fecal matrices, controlled robustness assessments (e.g., standard-addition/spike-and-recovery and defined interference panels) will be included in future studies to provide additional quantitative evidence of matrix tolerance.

## Figures and Tables

**Table 1:** Composition of the datasets used for ANN model development and validation for SCFAs quantification. The numbers of training and test cases are listed for the propionic/butyric acid model and the total SCFAs model; the number of real fecal samples included in each set is indicated in parentheses.

Propionic Acid and Butyric Acid	
**Set**	**Case**
Training set	72 (18 real samples)
Test set	30 (real samples)
**Total SCFAs**	
Training set	156 (30 real samples)
Test set	30 (real samples)

**Table 2:** Comparison of GC–MS measurements and ANN predictions for SCFAs quantification in human fecal samples (n = 30). Concentrations of total SCFAs, propionic acid, and butyric acid are reported in mM for GC–MS and ANN model. Values in parentheses denote the relative deviation between ANN predictions and GC–MS measurements.

	GC–MS			ANN Model		
NO.	Total SCFAs (mM)	Propionic Acid (mM)	Butyric Acid (mM)	Total SCFAs (mM)	Propionic Acid (mM)	Butyric Acid (mM)
1	7.54	1.53	1.25	7.50 ± 0.68 (+0.5%)	1.58 ± 0.12 (−3.3%)	1.26 ± 0.09 (−0.8%)
2	2.46	0.78	0.23	2.40 ± 0.18 (+0.4%)	0.76 ± 0.05 (+2.6%)	0.23 ± 0.01 (+0.0%)
3	4.00	1.29	0.43	4.07 ± 0.31 (−1.8%)	1.34 ± 0.10 (−3.9%)	0.44 ± 0.03 (−2.3%)
4	21.32	3.93	3.53	21.27 ± 1.84 (+0.2%)	3.90 ± 0.27 (+0.8%)	3.54 ± 0.25 (−0.3%)
5	5.48	1.47	0.70	5.57 ± 0.42 (−1.6%)	1.45 ± 0.11 (+1.4%)	0.69 ± 0.04 (+1.4%)
6	5.07	1.96	0.33	5.07 ± 0.38 (+0.0%)	1.99 ± 0.15 (−1.5%)	0.32 ± 0.02 (+3.0%)
7	8.32	2.22	1.28	8.61 ± 0.74 (−3.5%)	2.25 ± 0.18 (−1.4%)	1.25 ± 0.09 (+2.3%)
8	4.49	1.50	0.49	4.87 ± 0.36 (−8.5%)	1.50 ± 0.11 (+0.0%)	0.48 ± 0.03 (+2.0%)
9	9.35	2.88	1.25	9.38 ± 0.79 (−0.3%)	2.86 ± 0.21 (+0.7%)	1.23 ± 0.10 (+1.6%)
10	9.05	1.85	0.81	8.45 ± 0.62 (+6.6%)	1.80 ± 0.14 (+2.7%)	0.82 ± 0.06 (−1.2%)
11	6.02	1.61	0.44	5.98 ± 0.45 (+0.7%)	1.60 ± 0.12 (+0.6%)	0.40 ± 0.03 (+9.1%)
12	7.00	1.68	0.92	7.49 ± 0.58 (−7.0%)	1.67 ± 0.12 (+0.6%)	0.88 ± 0.06 (+4.3%)
13	6.79	1.97	1.03	6.96 ± 0.51 (−2.5%)	2.00 ± 0.16 (−1.5%)	0.95 ± 0.07 (+7.8%)
14	1.32	0.37	0.10	1.27 ± 0.09 (+3.8%)	0.34 ± 0.02 (+8.1%)	0.08 ± 0.01 (+20.0%)
15	6.22	0.58	0.94	6.06 ± 0.43 (+2.6%)	0.45 ± 0.03 (+22.4%)	0.92 ± 0.06 (+2.1%)
16	6.73	2.82	0.27	6.66 ± 0.48 (+1.0%)	2.86 ± 0.22 (−1.4%)	0.29 ± 0.02 (−7.4%)
17	5.32	1.26	0.34	5.42 ± 0.41 (−1.9%)	1.30 ± 0.09 (−3.2%)	0.35 ± 0.02 (−2.9%)
18	9.66	2.37	1.24	9.27 ± 0.81 (+4.0%)	2.34 ± 0.17 (+1.3%)	1.18 ± 0.08 (+4.8%)
19	5.64	1.38	0.29	5.62 ± 0.39 (+0.4%)	1.47 ± 0.11 (−6.5%)	0.30 ± 0.02 (−3.4%)
20	2.77	0.61	0.29	2.82 ± 0.21 (−1.8%)	0.58 ± 0.04 (+4.9%)	0.30 ± 0.02 (−3.4%)
21	10.40	3.49	0.55	10.49 ± 0.88 (−0.9%)	3.35 ± 0.26 (+4.0%)	0.54 ± 0.04 (+1.8%)
22	3.06	0.86	0.47	3.16 ± 0.24 (−3.3%)	0.86 ± 0.06 (+0.0%)	0.44 ± 0.03 (+6.4%)
23	4.87	1.08	0.65	4.87 ± 0.37 (+0.0%)	1.06 ± 0.08 (+1.9%)	0.65 ± 0.05 (+0.0%)
24	2.85	0.71	0.29	2.78 ± 0.20 (+2.5%)	0.68 ± 0.05 (+4.2%)	0.30 ± 0.02 (−3.4%)
25	7.55	2.03	0.79	7.62 ± 0.59 (−0.9%)	2.00 ± 0.15 (+1.5%)	0.76 ± 0.05 (+3.8%)
26	5.97	0.94	0.56	5.68 ± 0.42 (+4.9%)	0.90 ± 0.07 (+4.3%)	0.56 ± 0.04 (+0.0%)
27	6.41	1.71	1.09	6.38 ± 0.51 (+0.5%)	1.64 ± 0.12 (+4.1%)	1.09 ± 0.08 (+0.0%)
28	5.33	1.05	0.94	5.43 ± 0.38 (−1.9%)	1.07 ± 0.08 (−1.9%)	0.90 ± 0.06 (+4.3%)
29	9.07	1.80	1.15	8.96 ± 0.72 (+1.2%)	1.67 ± 0.13 (+7.2%)	1.08 ± 0.08 (+6.1%)
30	9.37	3.33	0.87	9.42 ± 0.83 (−0.5%)	3.35 ± 0.24 (−0.6%)	0.84 ± 0.06 (+3.4%)

**Table 3:** Performance comparison of different models for the quantification of propionic acid, butyric acid, and total SCFAs using the MLR, RF, and ANN models.

Propionic acid			
**Analysis model**	**MAE**	**RMSE**	**Accuracy (%)**
MLR model	0.494	0.417	20.1%
RF model	0.100	0.142	90%
ANN model (this study)	0.063	0.072	96.66%
**Butyric acid**			
**Analysis model**	**MAE**	**RMSE**	**Accuracy (%)**
MLR model	0.413	0.304	10%
RF model	0.059	0.100	66.66%
ANN model (this study)	0.029	0.034	96.66%
**Total SCFAs**			
**Analysis model**	**MAE**	**RMSE**	**Accuracy (%)**
MLR model	1.906	2.987	0.00%
RF model	0.655	0.689	43.3%
ANN model (this study)	0.135	0.202	90.0%

## Data Availability

The data presented in this study are not publicly available due to privacy and ethical restrictions associated with human fecal specimens and related clinical information. De-identified data supporting the findings of this study are available from the corresponding author upon reasonable request and subject to institutional approval.

## Supplementary Materials

The following supporting information can be downloaded at: https://www.mdpi.com/article/10.3390/bios16040223/s1, Section S1: Workflow time comparison (typical laboratory conditions). Section S2: Hyperparameter selection and architecture optimization for ANN regression. Table S1: Hyperparameter search space explored for ANN regression, including network size, regularization, optimizer settings, and training parameters for the propionate/butyrate and total SCFAs models. Table S2: Final selected ANN architectures and training settings for propionate/butyrate and total SCFAs regression. Figure S1: Electrochemical impedance spectroscopy (EIS) quality-control of disposable planar Au chips. Figure S2: ANN parity plots for internal training and test splits during model development. Figure S3: Representative voltammetric fingerprints from real human fecal samples. Figure S4: Bland–Altman agreement analysis between the ANN-assisted electrochemical workflow and GC–MS for propionic acid quantification in human fecal samples (n = 30). Figure S5: Bland–Altman agreement analysis between the ANN-assisted electrochemical workflow and GC–MS for butyric acid quantification in human fecal samples (n = 30). Figure S6: Bland–Altman agreement analysis between the ANN-assisted electrochemical workflow and GC–MS for total SCFAs quantification in human fecal samples (n = 30). Table S3: Method-comparison statistics between ANN predictions and GC–MS in the independent fecal test set (n = 30). Table S4: Comparison of representative electrochemical and rapid methods for SCFAs quantification.

## Abbreviations

The following abbreviations are used in this manuscript:

ANN	Artificial neural network
AI	Artificial intelligence
CV	Cyclic voltammetry
DPV	Differential pulse voltammetry
EIS	Electrochemical impedance spectroscopy
FID	Flame ionization detection
GC	Gas chromatography
GC–MS	Gas chromatography–mass spectrometry
IBD	Inflammatory bowel disease
IBS	Irritable bowel syndrome
MAE	Mean absolute error
ML	Machine learning
MLP	Multilayer perceptron
MLR	Multiple linear regression
MS	Mass spectrometry
PBS	Phosphate-buffered saline
ReLU	Rectified linear unit
RF	Random forest
RMSE	Root mean square error
SCFAs	Short-chain fatty acids
SPE	Screen-printed electrode
