# Supplementary material for: Rapid Electrochemical Profiling of Fecal Short-Chain Fatty Acids Using Esterification/Dissociation Fingerprints and Artificial Neural Networks
Source: Biosensors (Basel). 2026 Apr 17;16(4):223. doi: 10.3390/bios16040223 (PMC13114974; doi:10.3390/bios16040223)
Supplement: Supplementary file 1 [file biosensors-16-00223-s001.zip › biosensors-4216210-supplementary.pdf]

Section S1. Workflow time comparison (typical laboratory conditions).

In the proposed electrochemical–ANN workflow, pretreatment dominates the turnaround time for a single sample (propionate esterification ~3 h + 15 min neutralization; butyrate dissociation ~3 h; total-SCFAs esterification ~1 h). However, these pretreatment steps are batch-compatible and can be performed in parallel, so the batch turnaround is governed by the longest pretreatment (≈ 3.25 h) plus the minute-scale electrochemical acquisition (≤ 3 min per sample for DPV/CV readout of the three targets). Thus, for a batch of *N* samples, the total electrochemical time can be approximated as $T_{EC} \approx 3.25$ h + (3 min × N). By contrast, the GC–MS workflow typically requires ~3.5 h instrument startup/calibration, followed by ~40 min per sample analyzed sequentially ($T_{GC\text{-}MS} \approx 3.5$ h + 40 min × N). For example, for N = 30 samples, $T_{EC} \approx 4.75$ h, whereas $T_{GC\text{-}MS} \approx 23.5$ h, highlighting the throughput advantage of batch pretreatment combined with rapid per-sample electrochemical acquisition. These time estimates reflect typical laboratory conditions in this study and may vary with instrument setup and operating protocols.

Section S2. Hyperparameter Selection and Architecture Optimization for ANN Regression

To address concerns about model complexity and potential overfitting, this section describes the preprocessing, cross-validation strategy, and hyperparameter optimization used for the ANN regressors in Section 2.7. Both models were trained on low-dimensional engineered feature vectors (10 features for propionic/butyric acid prediction and 7 features for total SCFAs prediction). Final model settings were selected based on cross-validated stability and agreement between training and validation/test performance, rather than training accuracy alone.

S2.1. Data preprocessing and normalization

Because the extracted electrochemical descriptors span different numeric ranges, feature scaling was applied to improve convergence and numerical stability. Each feature was rescaled to [0, 1] using min–max normalization:

$$x_{normalized} = \frac{x - x_{\min}}{x_{\max} - x_{\min}} \quad \text{(S1-1)}$$

Here, x denotes the raw value of an electrochemical feature extracted from the DPV/CV fingerprints for propionic acid, butyric acid, and total SCFAs. For each feature dimension, $x_{\max}$and $x_{\min}$are defined as the maximum and minimum values observed within the training dataset for that feature, respectively; thus, normalization was performed feature-wise (each descriptor has its own $x_{\max}$and $x_{\min}$). To avoid data leakage, normalization parameters were computed exclusively from the training set and then applied unchanged to the validation/test sets using the training-set minima and maxima.

S2.2. Model development workflow and overfitting control

Model development followed three stages. First, Pearson correlation analysis was used to quantify linear associations between candidate electrochemical descriptors and SCFAs concentrations, supporting feature screening and interpretation. Second, ANN regression models were trained using the engineered feature vectors described in Section 2.7. Third, model robustness and overfitting risk were evaluated using five-fold cross-validation (k = 5) on the training data. In each fold, one subset served as the validation set and the remaining subsets were used for training; this procedure was repeated until all subsets had served once as validation. Model stability was assessed by examining fold-to-fold consistency of performance metrics (R^2, RMSE, MAE) and by inspecting training versus validation loss behavior. When pronounced fold-to-fold variance suggested possible overfitting or strong outlier influence (a common issue in fecal matrices), additional sensitivity checks were conducted by repeating validation with smaller holdout fractions (5-10%) to diagnose instability sources and refine model settings.

S2.3. Hyperparameter screening and selection criteria

Hyperparameters were then systematically varied within a predefined search space (Table S1), including the number of neurons, dropout rate, optimizer type, learning rate, loss function, batch size, and training iterations/epochs. The final configuration for each task was selected using combined criteria of: (i) improved predictive accuracy (lower RMSE/MAE and higher R^2), (ii) stable validation-loss behavior across folds (no divergence or instability), and (iii) consistency between training and validation/test performance. This selection strategy was adopted to ensure that improvements in training accuracy did not come at the expense of generalization. The hyperparameter search space is summarized in Table S1, and the final selected configurations are listed in Table S2.

S2.4. Final ANN configuration for propionate/butyrate regression

Based on the cross-validated screening procedure described above, the optimized configuration for propionate/butyrate regression employed a single hidden layer and was trained using a 10-feature input vector. The final setting was: 4000 neurons, ReLU activation, dropout = 0, Adam optimizer (learning rate 1×10^{-4}), Huber loss, batch size = 50, and 5000 epochs. This configuration provided the best balance between prediction accuracy and stability, with consistent training–validation behavior and reliable performance on the independent fecal test set.

S2.5. Final ANN configuration for total SCFAs regression

For total SCFAs regression, the same screening framework was applied using the 7-feature input vector (electrochemical descriptors plus auxiliary inputs derived from the propionate/butyrate models, as described in Section 2.7). The optimized configuration for the total SCFAs ANN employed a single hidden layer with 1000 neurons, ReLU activation, dropout = 0.1, Nadam optimizer (learning rate 1×10^{-4}), Huber loss, batch size = 30, and 5000 epochs. The inclusion of a small dropout term reflects the optimization outcome under cross-validation, improving generalization stability for the integrated regression task. The final selected configurations for both models are summarized in Table S2.

Table S1. Hyperparameter search space explored for ANN regression, including network size, regularization, optimizer settings, and training parameters for the propionate/butyrate and total SCFAs models.

Category	Values tested
Neurons (hidden layer)	500-6000
Dropout rate	0, 0.1, 0.2
Optimizer	Nadam, SGD, Adagrad, RMSprop, Adam
Learning rate	1×10^{-5}, 1×10^{-4}, 1×10^{-3}
Loss function	Huber, MSE, MAE, MAPE, MSLE, LogCosh
Training iterations/epochs	3000, 5000, 7000
Batch size	10, 30, 50, 90
Hidden layers	1 (screened as primary architecture)

Table S2. Final selected ANN architectures and training settings for propionate/butyrate and total SCFAs regression (input dimensionality, network size, regularization, optimizer/loss, and training schedule).

Item	Propionate/Butyrate ANN	Total SCFAs ANN
Input dimensionality	10 features	7 features
Hidden layers	1	1
Neurons (hidden layer)	4000	1000
Activation	ReLU	ReLU
Dropout	0	0.1
Optimizer	Adam	Nadam
Learning rate	1×10^{-4}	1×10^{-4}
Loss function	Huber	Huber
Batch size	50	30
Training iterations/epochs	5000	5000

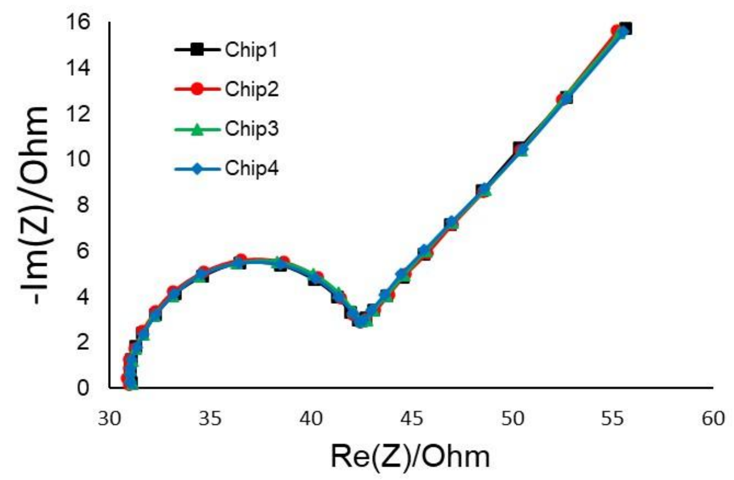

Figure S1. Electrochemical impedance spectroscopy (EIS) quality-control of disposable planar Au chips. Nyquist plots (−Im(Z) vs. Re(Z)) were recorded in a standard ferri/ferrocyanide redox-probe solution using four independently tested disposable Au chips (Chip1–Chip4) under identical conditions. The spectra closely overlap, indicating good chip-to-chip reproducibility. The high-frequency intercept yields a solution resistance on the order of tens of ohms ($R_s \approx 31\ \Omega$), implying an iR drop of <1 mV at the μA-level currents observed in the SCFA voltammetry and therefore negligible impact on the extracted fingerprint features.

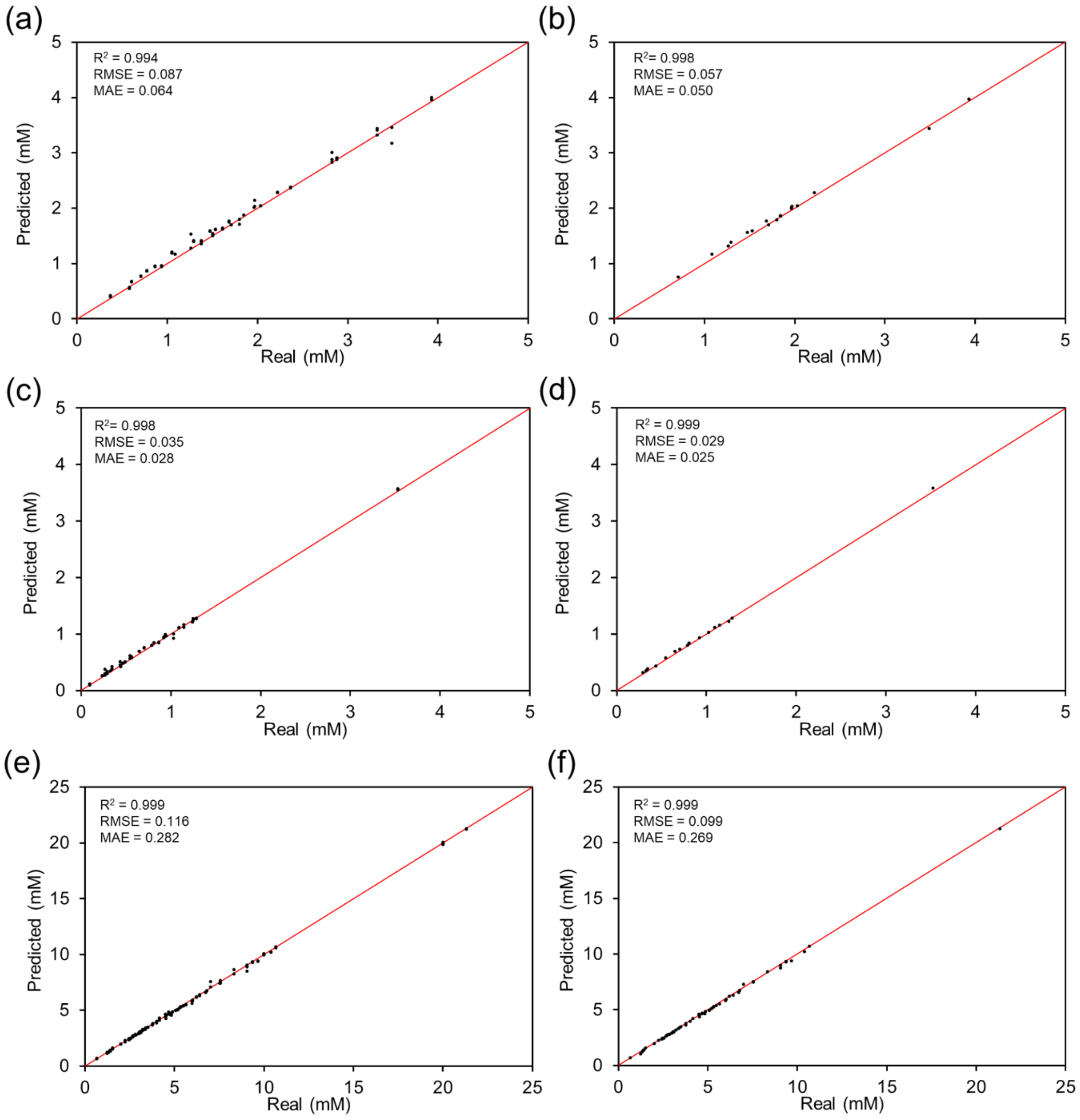

Figure S2. ANN parity plots for internal training and test splits during model development. Predicted concentrations are plotted against reference values for (a) propionic acid (training), (b) propionic acid (internal test), (c) butyric acid (training), (d) butyric acid (internal test), (e) total SCFAs (training), and (f) total SCFAs (internal test); the solid diagonal line denotes ideal 1:1 agreement. Independent fecal validation and method-comparison statistics versus GC–MS are reported in Fig. 5/Table 3 and Figs. S2–S4/Table S3.

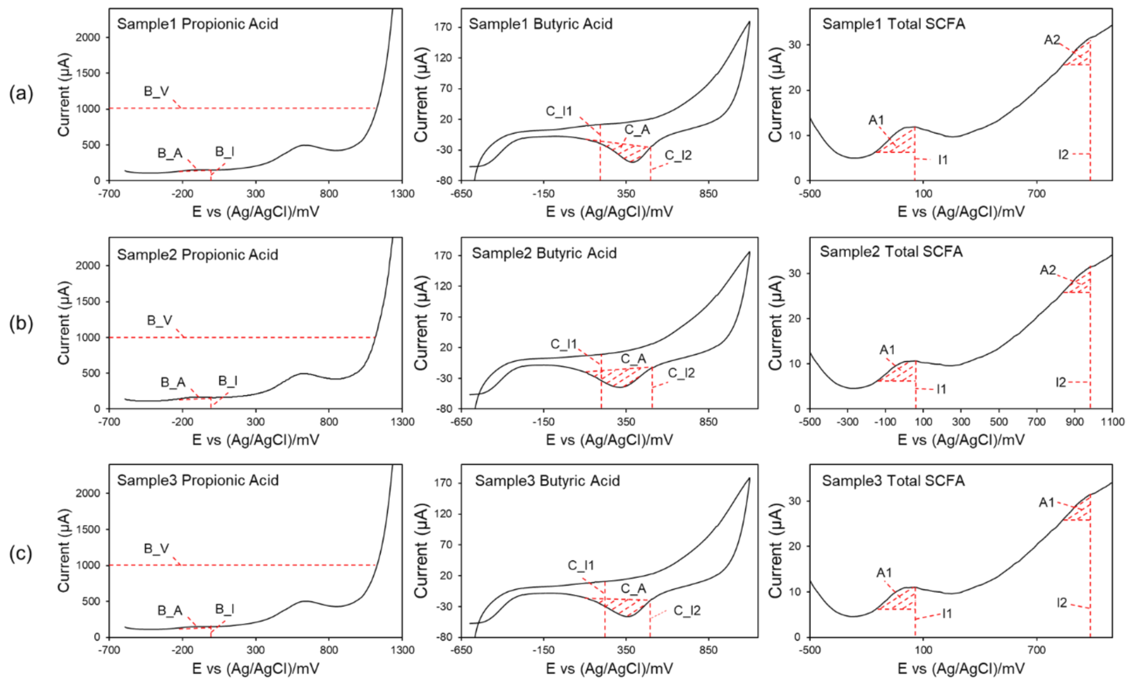

Figure S3. Representative voltammetric fingerprints from real human fecal samples. Differential pulse voltammetry (DPV) and cyclic voltammetry (CV) traces were recorded from three fecal specimens (Sample 1–Sample 3; panels a–c) after the corresponding pretreatment routes. Left column: DPV fingerprints for propionic acid after esterification pretreatment, with the extracted feature descriptors indicated (B_A, B_I, B_V). Middle column: CV fingerprints for butyric acid after alkaline dissociation pretreatment, highlighting the anodic current descriptor at 175 mV (C_I1)and auxiliary fingerprint descriptors (C_A, C_I2). Right column: DPV fingerprints for total SCFAs after esterification pretreatment, showing the two feature regions used for extraction (A_A1, A_A2 and corresponding current descriptors, A_I1, A_I2). Red dashed guides mark the baseline-corrected feature windows used for ANN inputs (Section 2.7). All potentials are referenced to the on-chip Ag/AgCl electrode (vs. Ag/AgCl), and each trace was recorded on a fresh single-use disposable Au SPE (first scan only).

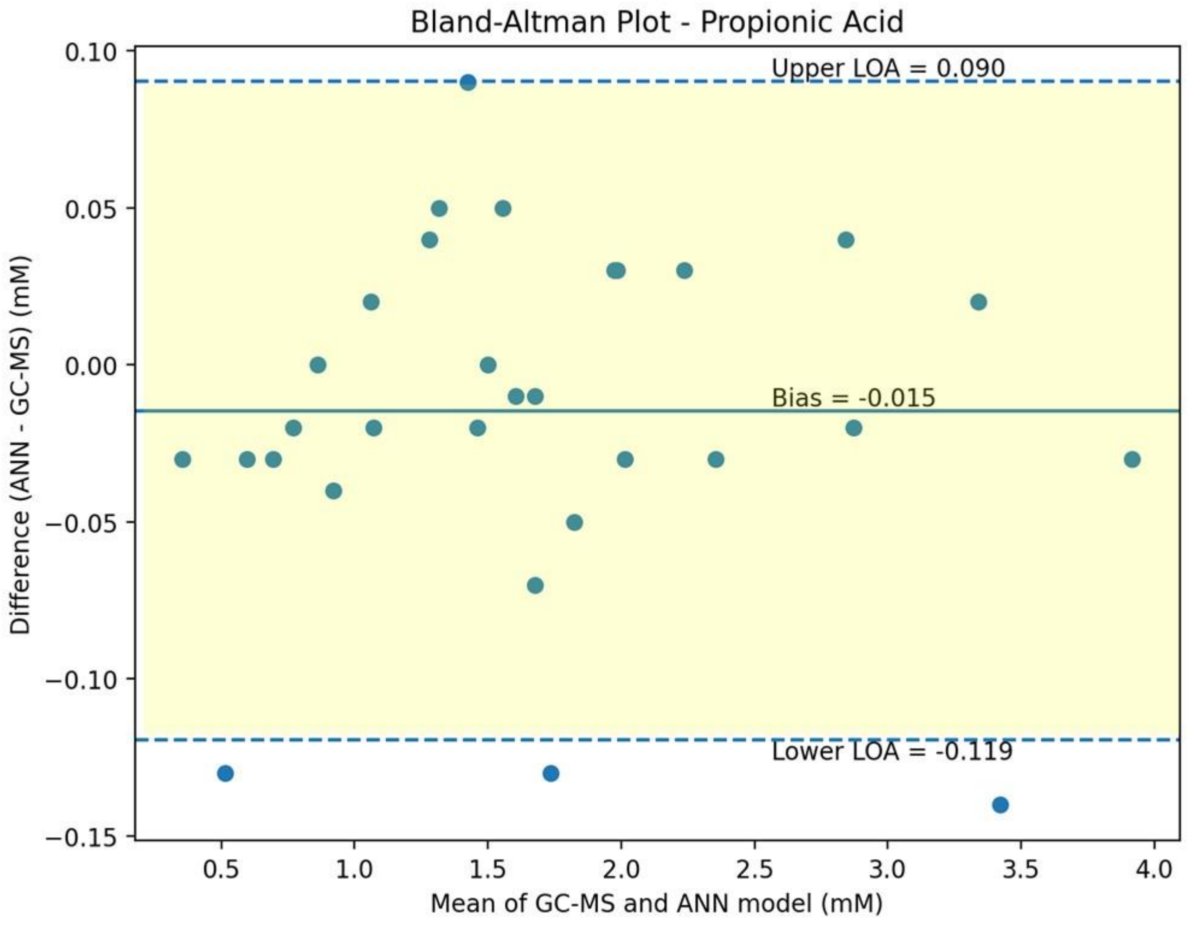

Figure S4. Bland–Altman agreement analysis between the ANN-assisted electrochemical workflow and GC–MS for propionic acid quantification in human fecal samples (n = 30). The y-axis shows the concentration difference (ANN − GC–MS), and the x-axis shows the mean of the two methods. The solid line denotes the mean bias (−0.015 mM), and the dashed lines denote the 95% limits of agreement (mean bias ± 1.96 SD; −0.119 to 0.090 mM).

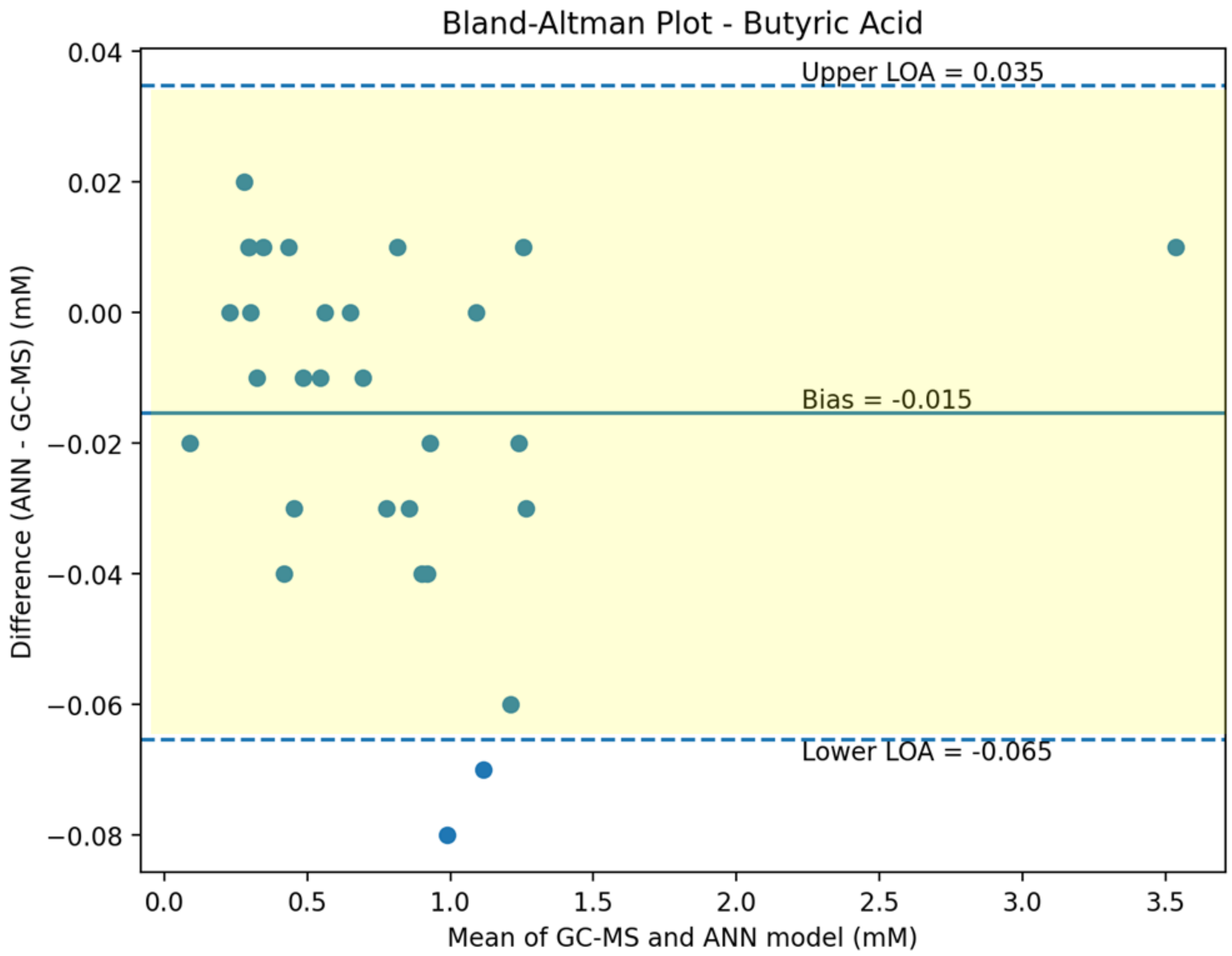

Figure S5. Bland–Altman agreement analysis between the ANN-assisted electrochemical workflow and GC–MS for butyric acid quantification in human fecal samples (n = 30). The y-axis shows the concentration difference (ANN − GC–MS), and the x-axis shows the mean of the two methods. The solid line denotes the mean bias (−0.015 mM), and the dashed lines denote the 95% limits of agreement (mean bias ± 1.96 SD; −0.065 to 0.035 mM).

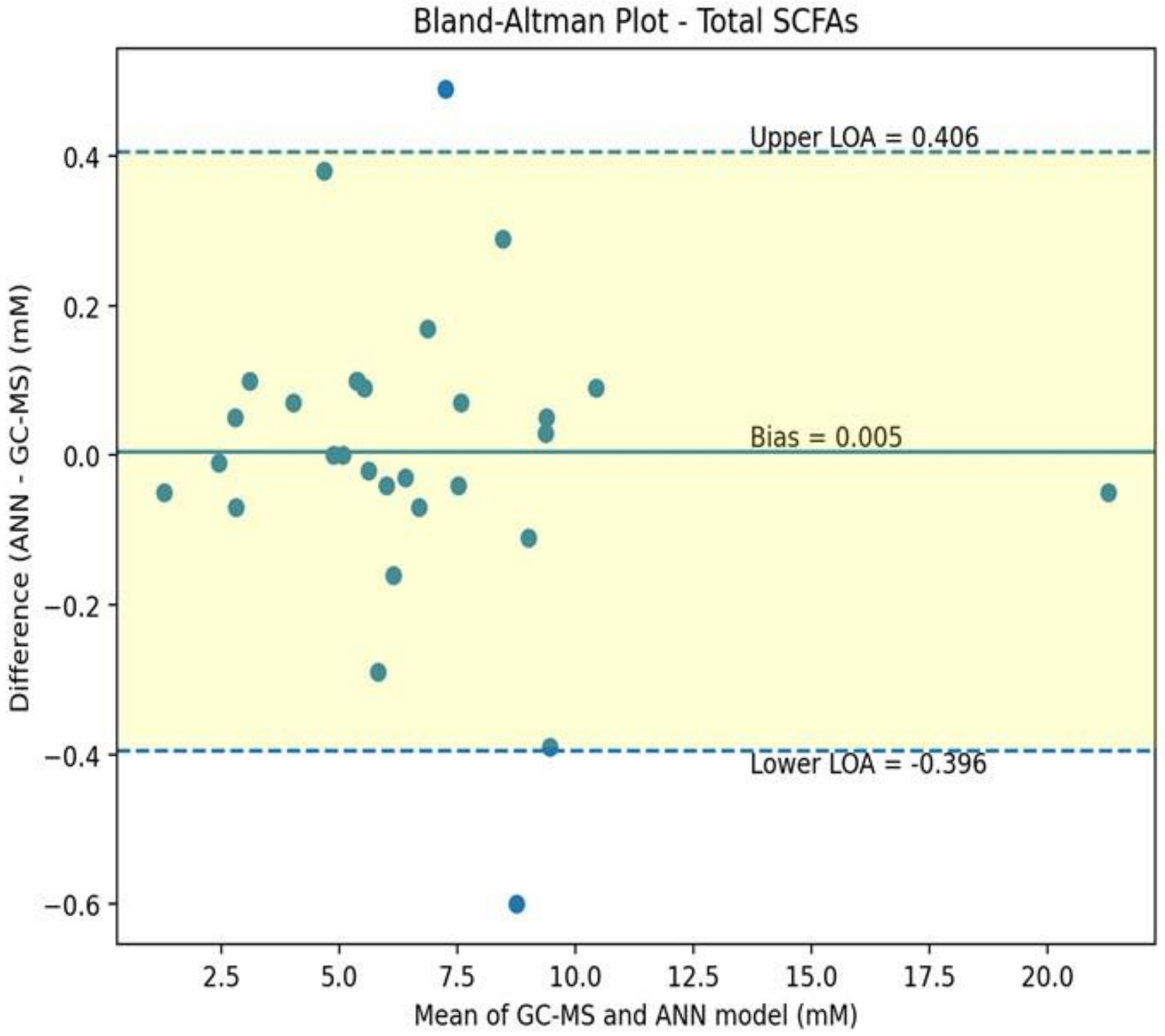

Figure S6. Bland–Altman agreement analysis between the ANN-assisted electrochemical workflow and GC–MS for total SCFAs quantification in human fecal samples (n = 30). The y-axis shows the concentration difference (ANN − GC–MS), and the x-axis shows the mean of the two methods. The solid line denotes the mean bias (0.005 mM), and the dashed lines denote the 95% limits of agreement (mean bias ± 1.96 SD; −0.396 to 0.406 mM).

Table S3. Method-comparison statistics between ANN predictions and GC–MS in the independent fecal test set (n = 30). Mean bias is defined as the paired difference (ANN − GC–MS). The 95% CI for mean bias was calculated as $CI = \bar{d} \pm t_{0.95,n-1} \times (\frac{s_d}{\sqrt{n}})$, where $\bar{d}$ is the mean bias, s_d is the standard deviation of paired differences and n is the sample size. Limits of agreement (LoA) were computed as$\bar{d} \pm 1.96 s_d$. RMSE is reported with a two-sided bootstrap 95% CI. Paired t-test and Wilcoxon signed-rank p-values test whether the paired differences have zero mean or zero median, respectively. Bootstrap CIs for RMSE were obtained by resampling paired observations with replacement (e.g., 10,000 iterations) and taking the 2.5th and 97.5th percentiles.

**Analyte**	**Mean bias (mM)**	**95% CI of mean bias (mM)**	**LoA lower**	**LoA upper**	**RMSE (mM)**	**95% CI of RMSE (mM)**	**Paired t-test p-value**	**Wilcoxon signed-rank p-value**
Propionic acid	−0.0150	−0.0349 to 0.0049	-0.119	0.090	0.072	0.0483 to 0.093	0.1439	0.2889
Butyric acid	−0.0150	−0.0245 to −0.0055	-0.065	0.035	0.034	0.0229 to 0.0443	0.0027	0.0031
Total SCFAs	0.005	−0.0714 to 0.0814	-0.396	0.406	0.202	0.1228 to 0.2710	0.8944	0.5768

Table S4. Comparison of representative electrochemical and rapid methods for SCFAs quantification. Performance metrics, sample preparation, and timing are reported as described in the cited references; "NR" = not reported. Instrument cost tier is a qualitative estimate (Low/Medium/High) to allow fair comparison across platforms. For batch-time and per-sample cost under our laboratory conditions, see Supplementary Section S1.

**Method / Reference**	**Target analytes**	**Sample matrix**	**Pretreatment**	**Readout / throughput**	**Portability**	**Instrument cost tier**	**Validation in authentic fecal samples**	**Key notes**
**This work (DPV/CV + ANN, dual pretreatment, disposable planar Au SPE)**	Propionic acid, butyric acid, total SCFAs	**Human feces**	Esterification (1–3 h) + dissociation (≈3 h), **batch-compatible**	**DPV/CV acquisition: minutes per sample**; batch pretreatment dominates	**Yes**	**Low–Medium**	**Yes (n = 30; benchmarked vs GC–MS)**	ANN suppresses fecal-matrix interference; multi-analyte profiling with orthogonal fingerprints
PEDOT-coated electrode, pulsed/amperometric quantification [1]	Short-chain carboxylic acids / VFAs	Aqueous/controlled matrices	NR / limited	Rapid electrochemical readout (reported)	Potentially portable (depends on potentiostat)	Low–Medium	Not reported for human feces	Demonstrates feasibility/sensitivity in controlled solutions; limited complex-matrix validation
EIS chemiresistor (ZnO/PVA), SCFAs mixtures [2]	Multiple SCFAs (mixtures)	Electrolyte / complex media; bacterial culture applications	Dilution/handling per study; EIS-based	Demonstrated across physiologically relevant ranges (reported); mixture modeling	Miniaturized sensor concept	Medium	Not human feces; validated vs MS for bacterial secretion screening	Focuses on real-time monitoring in liquid phase; method differs from voltammetric fingerprinting
pDART-MS after amidation derivatization [3]	Multiple SCFAs	Various biological samples incl. feces (reported)	Derivatization (required)	**≈1 minute per sample (reported)**	MS platform (not portable)	**High**	Yes (reported in study context)	Very fast measurement but requires MS instrumentation and derivatization
HPLC-ECD [4]	SCFAs	Rat/human feces	Chromatographic workflow	Minutes-to-tens of minutes per run (typical HPLC)	No	High	Yes	Reliable laboratory method; throughput limited by chromatography; non-portable

Cost tier reflects typical instrument/access requirements (portable potentiostat < benchtop HPLC/EIS < MS-based systems). Reported readout times may not include sample pretreatment; readers should refer to Supplementary Section S1 for end-to-end workflow timing under typical laboratory conditions.

References

1. Feito, R.F.; Dinsdale, R.M.; Guwy, A.J.; Premier, G.C. Applicability of a PEDOT coated electrode for amperometric quantification of short chain carboxylic acids. *Sens. Actuators B Chem.* **2018**, *255*, 712–719.
2. Yavarinasab, A.; Flibotte, S.; Liu, S.; Tropini, C. An impedance-based chemiresistor for the real-time, simultaneous detection of gut microbiota-generated short-chain fatty acids. *Sens. Actuators B Chem.* **2023**, *393*, 134182.
3. Weng, C.Y.; Kuo, T.H.; Chai, L.M.X.; Zou, H.B.; Feng, T.H.; Huang, Y.J.; Tsai, J.C.; Wu, P.-H.; Chiu, Y.W.; Lan, E.I.; et al. Rapid quantification of gut microbial short-chain fatty acids by pDART-MS. Anal. Chem. 2020, 92, 14892–14897.
4. Kotani, A.; Miyaguchi, Y.; Kohama, M.; Ohtsuka, T.; Shiratori, T.; Kusu, F. Determination of short-chain fatty acids in rat and human feces by high-performance liquid chromatography with electrochemical detection. Anal. Sci. 2009, 25, 1007–1011.
